# Reconciliation of health records following penicillin allergy testing of hospitalized patients

**DOI:** 10.1186/1710-1492-10-S2-A39

**Published:** 2014-12-18

**Authors:** Rebecca Pratt, Anna Romanova, Joseph Greenbaum, Michael Cyr

**Affiliations:** 1 Department of Medicine, Division of Clinical Immunology and Allergy, McMaster University, Hamilton, Ontario, Canada; 2 Department of Medicine, University of Ottawa, Ottawa, Ontario, Canada

## Background

Medication errors are common and can lead to substantial morbidity. Similarly, inaccurate medication allergy lists can result in increased costs to the system, unnecessary allergy testing, prescription of inappropriate antibiotics, and allergic reactions. We hypothesized that most inpatients tested for penicillin allergy were not allergic and this information was not documented in the EMR or communicated to general practitioners.

## Methods

We retrospectively reviewed charts of all inpatients seen at a teaching hospital by a consultant allergist in 2012. Data collected included basic demographics, penicillin allergy test results, current allergy status in the EMR, readmission rates, prescribed antibiotics, and discharge summary contents.

## Results

146 patients were tested for penicillin allergy and 144 (98.6%) were not allergic. Although orders were written in 145 (99.3%) charts to update the allergy status after testing, 32 (22.23%) patients with negative tests were still listed as allergic to penicillin in the EMR. Only 19 (15.2%) discharge summaries notified family physicians of the allergy testing results and discharge summaries were missing for 25 (20%) patients. Further assessment of half the charts revealed that in 41% of cases the negative allergy test resulted in a change of antibiotic to penicillin or its derivative. Of the 60 readmitted patients, 20 (33%) were still listed as allergic to penicillin in the EMR (only one patient tested positive) and 14 (70%) of the 20 patients required antibiotics. 12 of these 14 patients (86%) were prescribed antibiotics in the penicillin family despite their positive allergy status.

## Conclusions

A significant proportion of health records were not amended following antibiotic allergy testing and the new allergy status was not communicated to most general practitioners in the discharge summary. A more efficient and reliable system needs to be implemented to ensure allergy status changes are communicated to all members of the healthcare team.

